# Identification of hub genes and pathways in adrenocortical carcinoma by integrated bioinformatic analysis

**DOI:** 10.1111/jcmm.15102

**Published:** 2020-03-08

**Authors:** Jinshuai Guo, Yinzhong Gu, Xiaoyu Ma, Lu Zhang, Huimin Li, Zhongyi Yan, Yali Han, Longxiang Xie, Xiangqian Guo

**Affiliations:** ^1^ Department of Predictive Medicine Institute of Biomedical Informatics Cell Signal Transduction Laboratory Bioinformatics Center Henan Provincial Engineering Center for Tumor Molecular Medicine School of Basic Medical Sciences Henan University Kaifeng China; ^2^ Education Bureau of Longgang District Shenzhen China

**Keywords:** adrenocortical carcinoma, Gene Expression Omnibus, GEPIA, overall survival, prognostic

## Abstract

Adrenocortical carcinoma (ACC), a rare malignant neoplasm originating from adrenal cortical cells, has high malignancy and few treatments. Therefore, it is necessary to explore the molecular mechanism of tumorigenesis, screen and verify potential biomarkers, which will provide new clues for the treatment and diagnosis of ACC. In this paper, three gene expression profiles (GSE10927, GSE12368 and GSE90713) were downloaded from the Gene Expression Omnibus (GEO) database. Differentially expressed genes (DEGs) were obtained using the Limma package. Gene ontology (GO) and Kyoto Encyclopedia of Genes and Genomes (KEGG) pathways were enriched by DAVID. Protein‐protein interaction (PPI) network was evaluated by STRING database, and PPI network was constructed by Cytoscape. Finally, GEPIA was used to validate hub genes’ expression. Compared with normal adrenal tissues, 74 up‐regulated DEGs and 126 down‐regulated DEGs were found in ACC samples; GO analysis showed that up‐regulated DEGs were enriched in organelle fission, nuclear division, spindle, et al, while down‐regulated DEGs were enriched in angiogenesis, proteinaceous extracellular matrix and growth factor activity; KEGG pathway analysis showed that up‐regulated DEGs were significantly enriched in cell cycle, cellular senescence and progesterone‐mediated oocyte maturation; Nine hub genes (*CCNB1, CDK1, TOP2A, CCNA2, CDKN3, MAD2L1, RACGAP1, BUB1* and *CCNB2*) were identified by PPI network; ACC patients with high expression of 9 hub genes were all associated with worse overall survival (OS). These hub genes and pathways might be involved in the tumorigenesis, which will offer the opportunities to develop the new therapeutic targets of ACC.

## 1 INTRODUCTION

Adrenal gland is an important endocrine organ of the human body and is also one of the most common organs with high tumour metastasis rate. According to the latest edition of pathological and genetic classification criteria of adrenal tumours published by World Health Organization (WHO), adrenal tumours can be divided into five categories: adrenocortical tumour, adrenal medullary tumour, extra‐adrenal paraganglioma, secondary tumour and other adrenal tumour.^1^ Adrenocortical tumour mainly includes adrenocortical adenoma (ACA) and adrenocortical carcinoma (ACC).^2^ ACC is a rare malignant tumour originating from adrenal cortical cells,^3^ and its incidence ranges from 0.7/10 00 000 to 2.0/10 00 000, which is under a high degree of malignancy, aggressiveness, high recurrence rate and poor prognosis. Most of the patients are found to have metastases and relapse easily after treatment, and the overall 5‐year survival rate was <35%.^4^ Early and accurate diagnosis is particularly important for the treatment and prognosis of ACC.^5^ At present, surgical resection is the only feasible method to cure ACC, but it is difficult to control its quality.^6^ Therefore, identifying new therapeutic targets or biomarkers for prognosis, diagnosis or prediction of ACC is urgently needed.

In recently years, many microarray profiling studies have been performed in ACC,^7^, ^8^ and hundreds of differentially expressed genes (DEGs) have been obtained. However, the results are limited or inconsistent due to molecular heterogeneity, and the results are usually generated from a single cohort study. Until now, no reliable biomarkers have been used in ACC clinics. Hence, the bioinformatics methods integrating multi‐cohorts analysed by gene microarray or RNAseq will be innovative and valuable for ACC research.

In this work, we downloaded three different Gene Expression Omnibus (GEO) datasets (GSE10927, GSE12368 and GSE90713) and screened differentially DEGs using the Limma package. Then, the PPI network in STRING database was constructed to screen the hub genes and pathways, and GEPIA database was used to verify hub genes and potential pathways. This study will offer the opportunities to develop the new therapeutic targets of ACC.

## 2 MATERIALS AND METHODS

**Figure 1:**
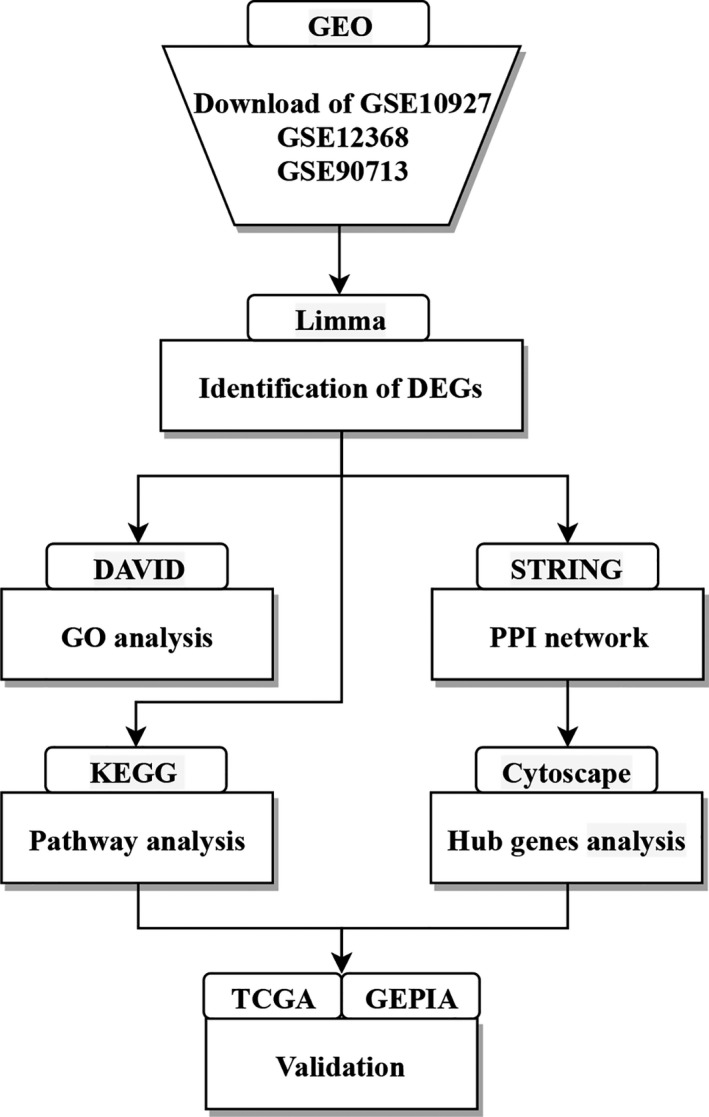
The flow diagram of this study

The flow diagram of this study was shown in Figure 1. The raw expression data were operated through a series of databases and software. This study has been proved by the Henan University institutional committee.

### 2.1 Data collection

Gene expression profiles of GSE10927,^9^ GSE12368^10^ and GSE90713^11^ were obtained from GEO database. The GSE10927 dataset includes 55 neoplastic samples and 10 non‐neoplastic samples (33 cases of ACC, 22 cases of ACA and 10 cases of normal adrenal cortex). The GSE12368 dataset is composed of 28 neoplastic samples and 6 non‐neoplastic samples (12 cases of ACC, 16 cases of ACA and 6 cases of normal adrenal cortex). The GSE90713 dataset includes 58 neoplastic samples and 5 non‐neoplastic samples (58 cases of ACC and 5 cases of normal adrenal cortex).

### 2.2 Screening differentially expressed genes

DEGs between ACC samples and non‐neoplastic samples were screened by using Limma package based on R language. DEGs were defined as representing differences with |log_2_FC| > 1, *P* < .05.^12^

### 2.3 Gene ontology and KEGG pathway enrichment analysis of DEGs

Gene ontology (GO) analysis annotates genes and gene products with functions of molecular function (MF), biological process (BP) and cellular component (CC).^13^, ^14^ Kyoto Encyclopedia of Genes and Genomes (KEGG) includes a series of genomics and enzymology methods and an online database of biochemical energy.^15^ The Database for Annotation, Visualization and Integrated Discovery (DAVID) (https://david.ncifcrf.gov/) is an online program providing a comprehensive set of functional annotation tools for investigators to understand biological meaning behind large list of genes.^16^ We performed GO terms and KEGG pathway analysis of DEGs by using DAVID database.

### 2.4 PPI network construction and hub module selection

Search Tool for the Retrieval of Interacting Genes (STRING) database (http://www.string-db.org/) was used to evaluate protein‐protein interaction (PPI).^17^ In addition, the database was used to quantify the relationships among the DEGs. Then, we used Cytoscape software to construct PPI network.^18^ The genes with the highest node score and the strongest connectivity were selected. *P* < .05 was considered to have statistical significance.

### 2.5 Hub genes validation

GEPIA (http://gepia.cancer-pku.cn/) is a powerful interactive web server that can analyse the RNA sequencing expression data of 9736 tumours and 8587 normal samples from the TCGA and the GTEx projects.^19^ GEPIA can be directly used for tumour/normal differential expression analysis according to cancer types, and the box plot will be shown to visualize the relationship. GEPIA was used to verify the hub genes and perform validation, *P* < .05 showed statistical significance.^19^

TCGA‐ACC RNA sequencing data with patient survival data were downloaded from the University of California, Santa Cruz (UCSC) Xena browser.^20^ Clinicopathological parameters of ACC patients with primary tumours, including age at diagnosis, gender, pathologic stage, living status, and overall survival (OS), were used for survival‐curve analysis.

### 2.6 Statistical analyses

Clinicopathologic parameter association analysis, and univariate and multivariate Cox regression analysis of 9 hub genes were performed with SPSS 22.0. Statistical significance was set at probability values of *P* < .05.

## 3 RESULTS

### 3.1 Identification of differentially expressed genes

**Figure 2:**
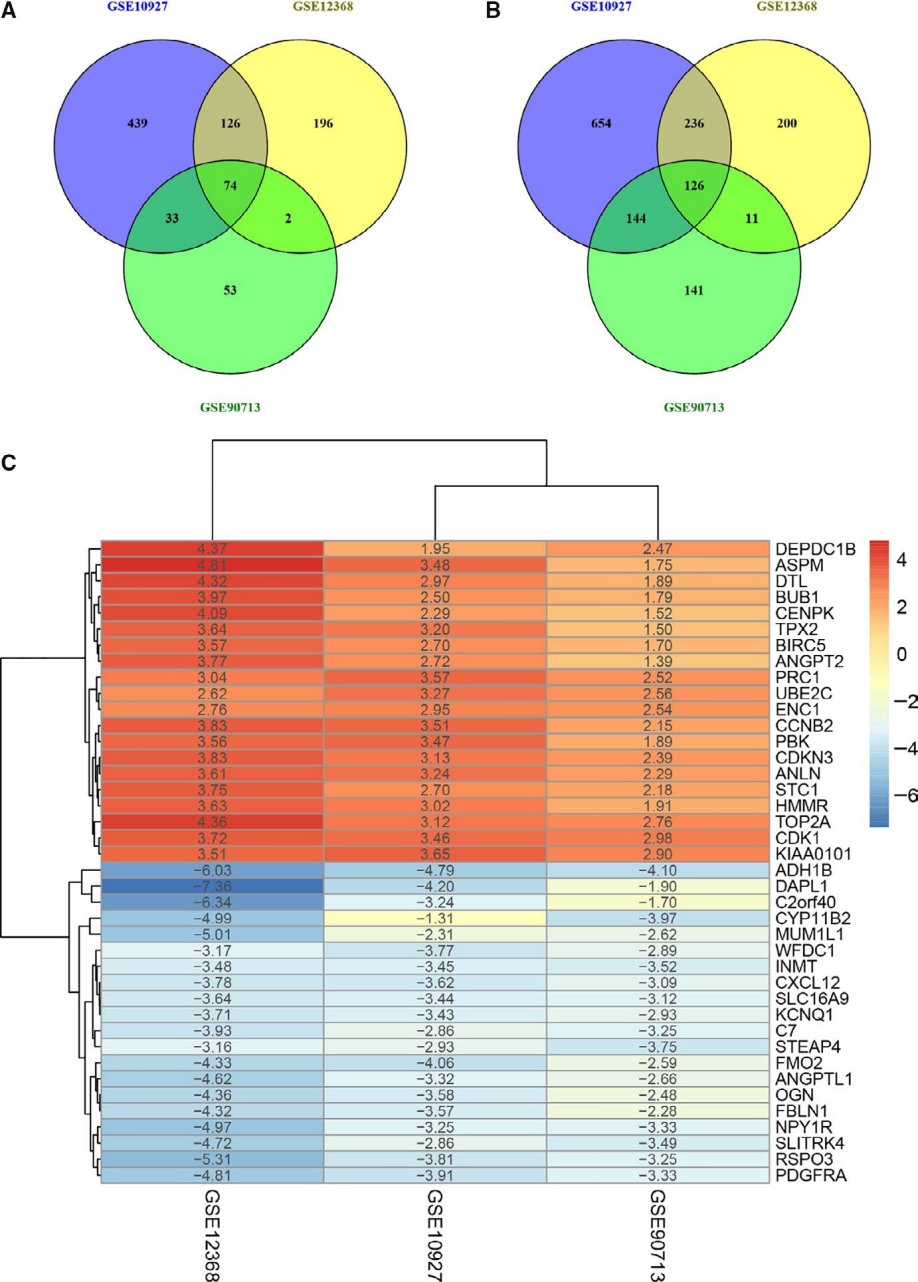
Venn diagram from intersection of differentially expressed genes (DEGs) in the three Gene Expression Omnibus (GEO) datasets and the heat map of top 40 DEGs. A, Up‐regulated genes in ACCs. B, Down‐regulated genes in ACCs. C, Heat map of top 40 representative DEGs. Blue represents a lower expression level, and red represents higher expression level

Using *P* < .05 and |log_2_FC| > 1 as cut‐off, we identified 1040 up‐regulated and 1821 down‐regulated genes in ACCs compared with normal tissues from GSE10927 dataset (Figure S1A), 2295 up‐regulated and 3300 down‐regulated genes from GSE12368 dataset (Figure S1B), 487 up‐regulated and 1129 down‐regulated genes from GSE90713 dataset (Figure S1C). By intersecting the DEGs across three datasets, a total of 200 consistently differentially expressed genes including 74 up‐regulated and 126 down‐regulated genes were identified to be significant in all the three above gene expression profiles (Figure 2A and B). The heat map of top 20 down‐regulated genes and top 20 up‐regulated genes’ expression was shown in Figure 2C.

### 3.2 Gene ontology analysis of differentially expressed genes

**Figure 3:**
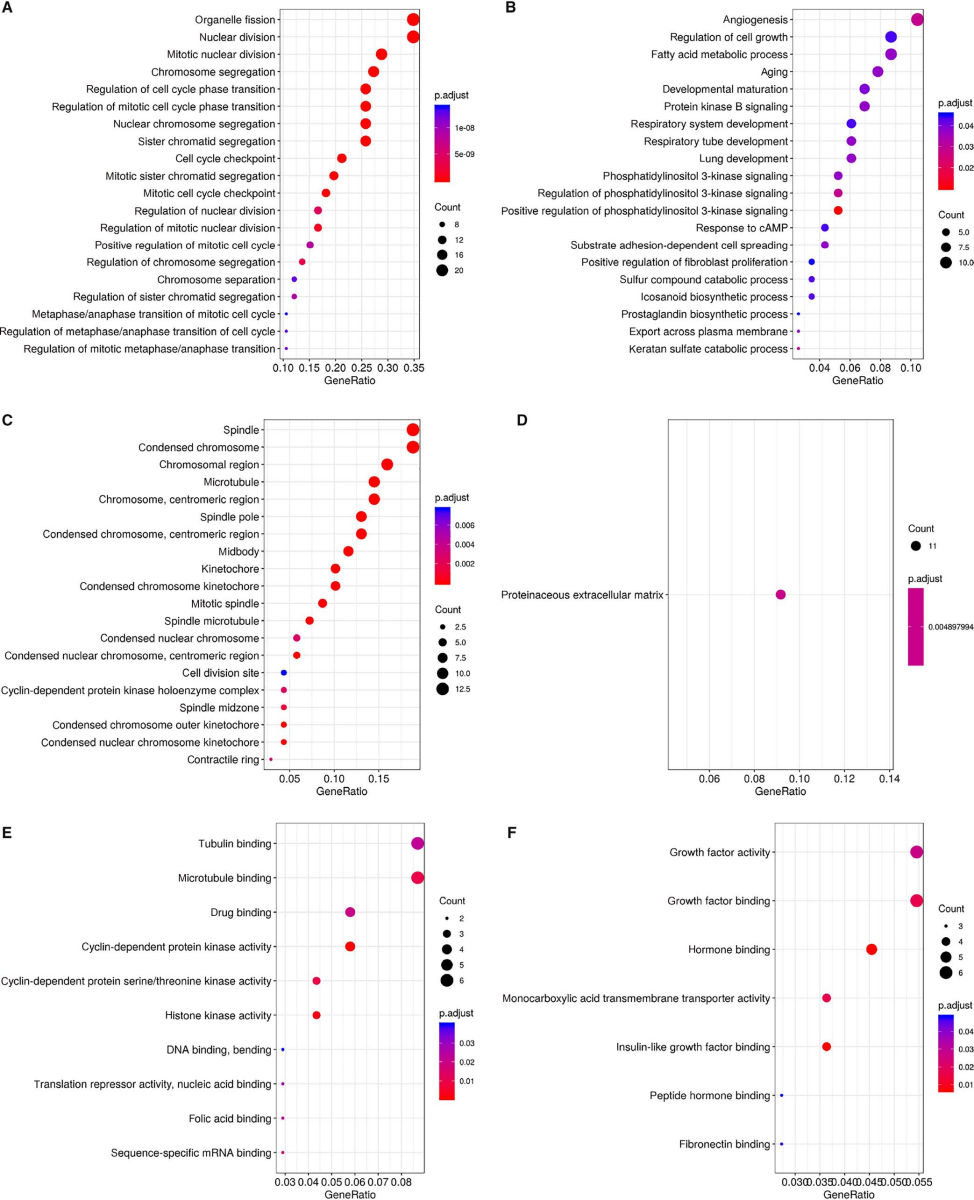
Gene ontology analysis of differentially expressed genes (DEGs) with |log_2_FC| > 1, *P* < .05. A, Biological process terms for up‐regulated DEGs. B, Biological process terms for down‐regulated DEGs. C, Cellular component terms for up‐regulated DEGs. D, Cellular component terms for down‐regulated DEGs. E, Molecular function terms for up‐regulated DEGs. F, Molecular function terms for down‐regulated DEGs

Subsequently, GO analysis of up‐regulated and down‐regulated DEGs was carried out by using DAVID online analysis tool.^16^ In terms of biological process (BP), up‐regulated DEGs were significantly enriched organelle fission, nuclear division, mitotic nuclear division, chromosome segregation, regulation of cell cycle phase transition, regulation of mitotic cell cycle phase transition, nuclear chromosome segregation and sister chromatid segregation (Figure 3A). Down‐regulated DEGs were involved in angiogenesis, regulation of cell growth, fatty acid metabolic process, ageing, developmental maturation and protein kinase B signalling (Figure 3B). In terms of cellular component (CC), the up‐regulated DEGs were significantly enriched in spindle, condensed chromosome, chromosomal region, microtubule, chromosome and centromeric region (Figure 3C), while the down‐regulated DEGs were significantly enriched in proteinaceous extracellular matrix (Figure 3D). In terms of molecular function (MF), up‐regulated DEGs were significantly enriched in tubulin binding, microtubule binding, drug binding and cyclin‐dependent protein kinase activity (Figure 3E), while the down‐regulated DEGs were significantly enriched in growth factor activity, growth factor binding and hormone binding (Figure 3F).

### 3.3 KEGG pathway enrichment analysis of differentially expressed genes

**Figure 4:**
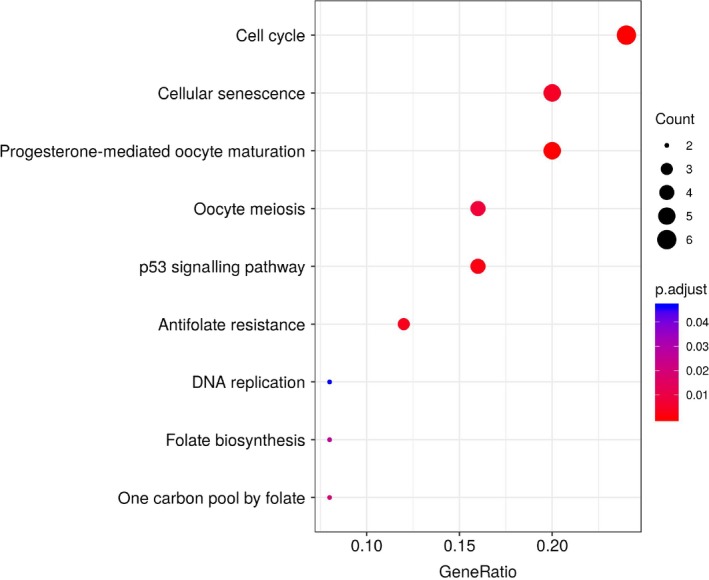
Significantly enriched pathway terms of up‐regulated differentially expressed genes in ACC

KEGG pathway analysis of all DEGs showed that most of up‐regulated DEGs were enriched in cell cycle, cell senescence, progesterone‐mediated oocyte maturation, oocyte, p53 signalling pathway and folic acid resistance (Figure 4), while down‐regulation of DEGs did not significantly enrich KEGG pathway.

### 3.4 PPI network construction and hub gene selection

**Figure 5:**
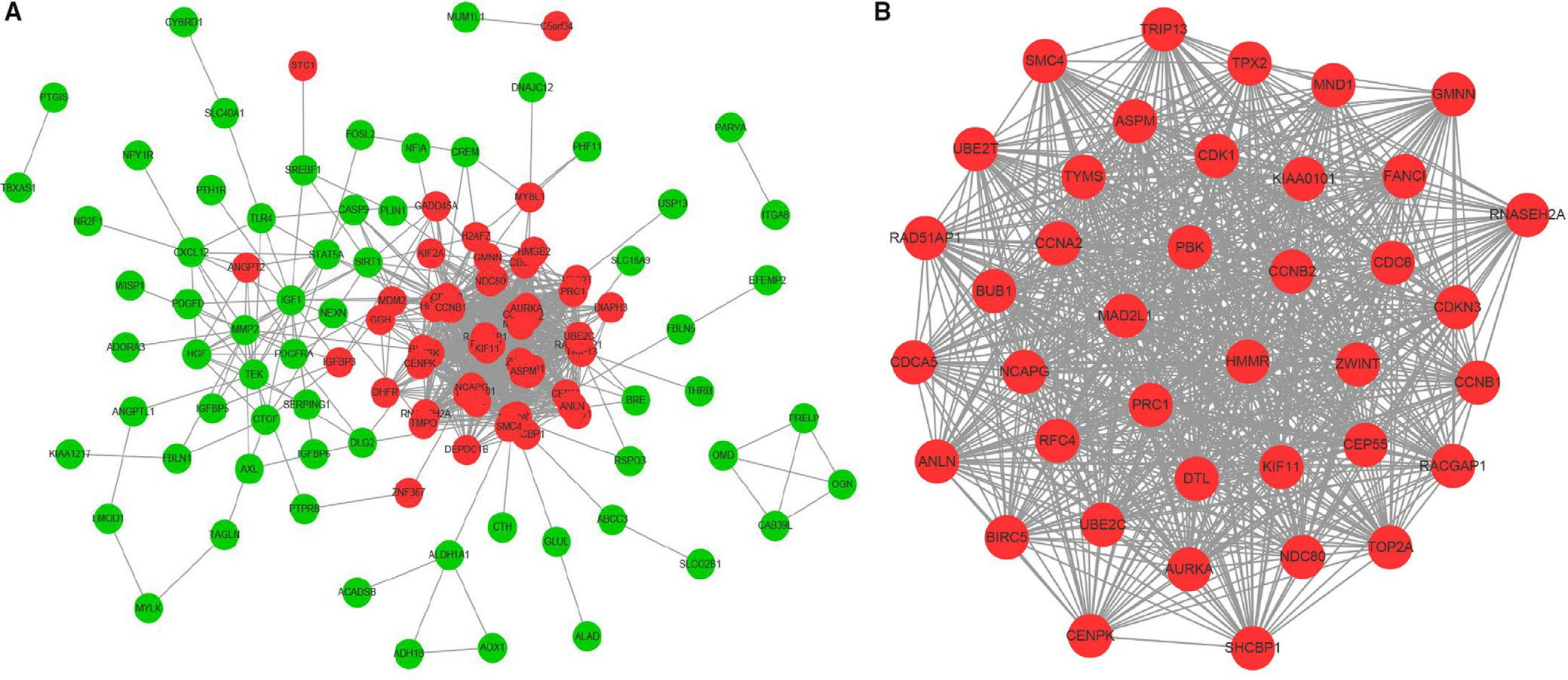
Protein‐protein interaction (PPI) network, module analysis and hub gene identification. Red nodes represent up‐regulated genes. Green nodes represent down‐regulated genes. A, PPI network of differentially expressed genes was constructed in STRING database. B, Top nine hub genes were selected by Cytoscape software based on the degree of each node

Through analysing STRING database^17^ and constructing PPI network by Cytoscape software,^18^ as shown in Figure 5, the PPI network of DEGs consists of 120 nodes and 1032 edges with the highest degree of 53, including 56 up‐regulated genes and 64 down‐regulated genes. It was considered that top nine DEGs with high degree of connectivity as the hub genes of ACC: *CCNB1* (Cyclin B1)*, CDK1* (cyclin‐dependent kinase 1)*, TOP2A* (topoisomerase IIα)*, CCNA2* (CyclinA2), *CDKN3* (cyclin‐dependent kinase inhibitor 3)*, MAD2L1* (mitosis arrest‐deficient 2 like 1)*, RACGAP1* (Rac GTPase activating protein 1)*, BUB1* (benzimidazole 1 homolog beta) and *CCNB2* (Cyclin B2). In terms of biological process, these hub genes are significantly enriched in mitotic spindle assembly checkpoint (Table S1). In terms of molecular function, these hub genes are significantly enriched in ATP binding (Table S1). KEGG pathway enrichment analysis showed that these hub genes are associated with progesterone‐mediated oocyte maturation, cell cycle, oocyte meiosis, p53 signalling pathway and progesterone‐mediated oocyte maturation (Table S1).

### 3.5 Evaluate the prognostic value of hub genes

**Figure 6:**
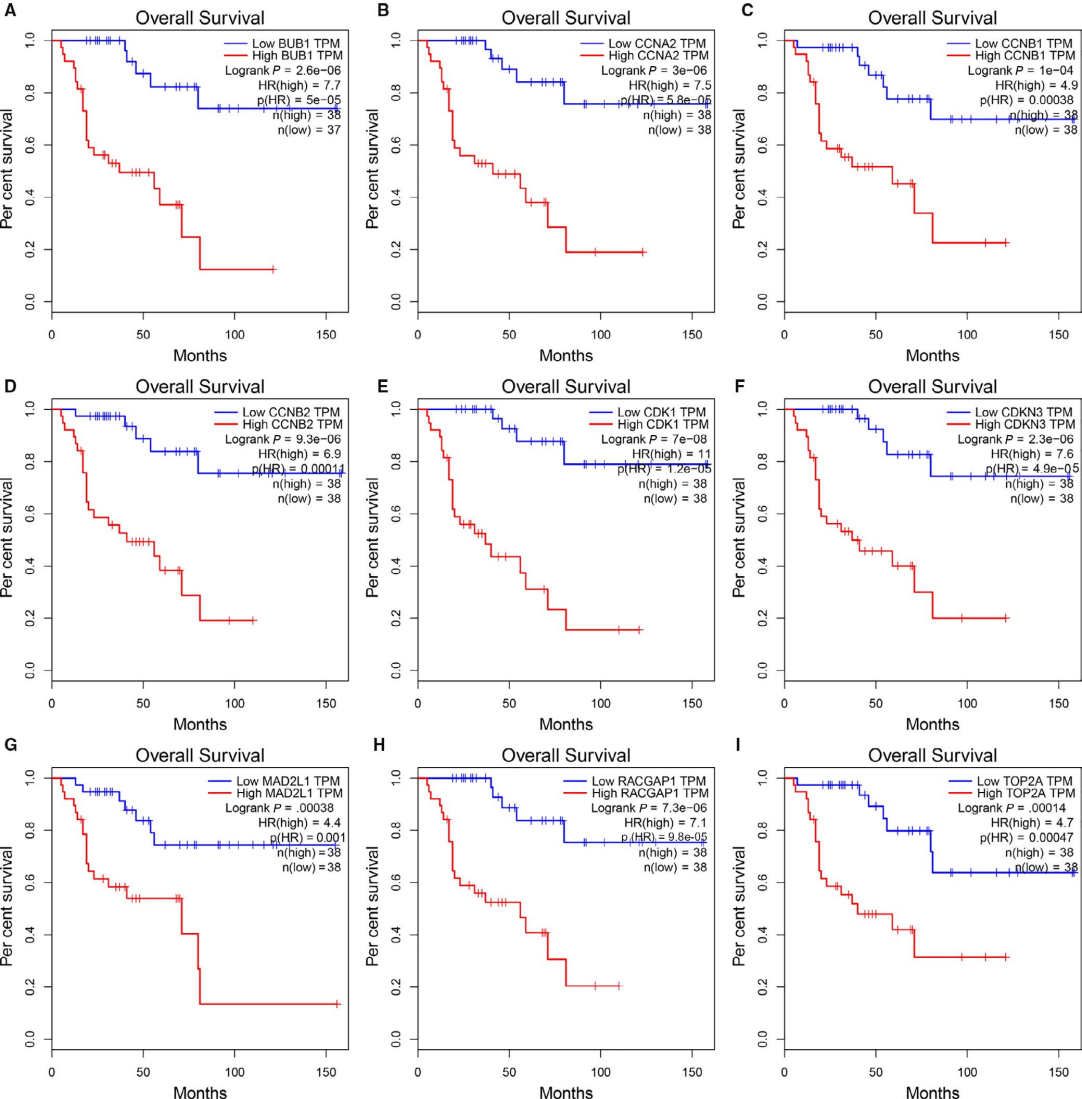
Prognostic value of 9 hub genes in ACCs by GEPIA. A, *BUB1*. B, *CCNA2*. C, *CCNB1.* D, *CCNB2*. E, *CDK1*. F, *CDKN3*. G, *MAD2L1*. H, *RACGAP1*. I, *TOP2A* *P* < .05 was as statistically significant

**Figure 7:**
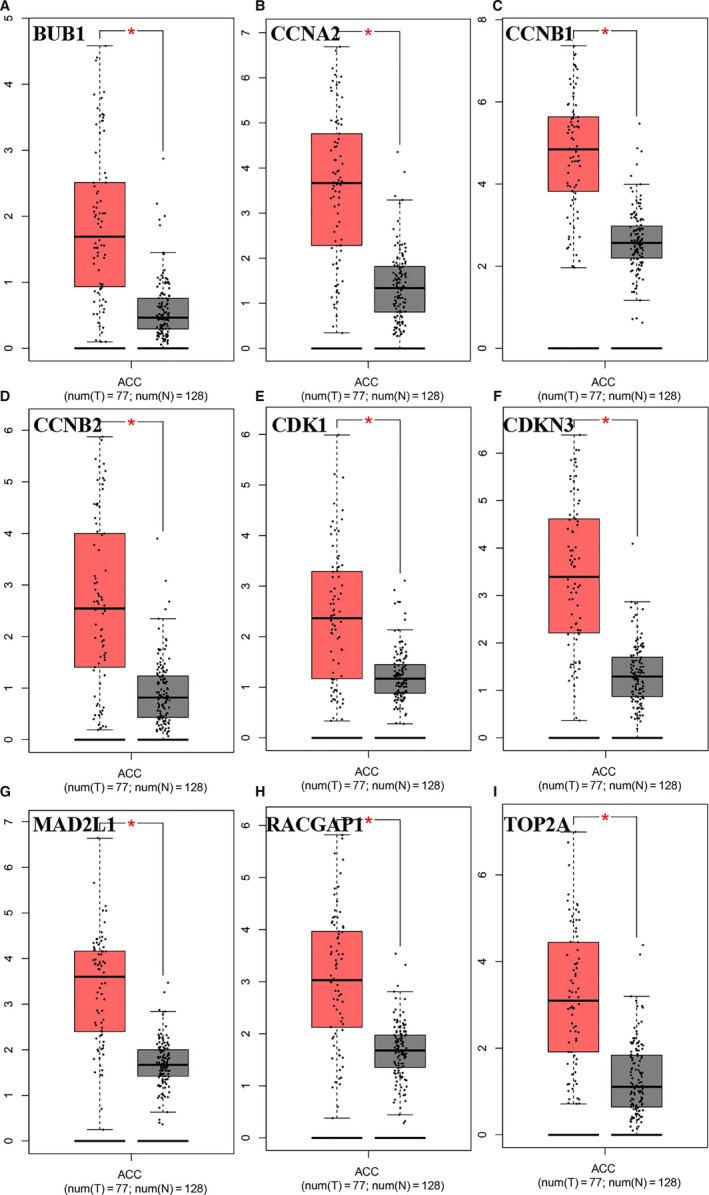
Nine hub genes are highly expressed in ACC tissues compared with normal tissues in GEPIA. The red and grey boxes represent cancer and normal tissues, respectively. A, *BUB1*. B, *CCNA2*. C, *CCNB1.* D, *CCNB2*. E, *CDK1*. F, *CDKN3*. G, *MAD2L1*. H, *RACGAP1*. I, *TOP2A* *P* < .05 was as statistically significant

TCGA‐ACC dataset was used to evaluate the prognostic value of nine hub genes by GEPIA. All patients with high hub gene expression were associated with worse OS (Figure 6). The additional univariate and multivariate Cox regression analysis showed that the hub gene *BUB1* (budding uninhibited by benzimidazole 1) was an independent prognostic factor for ACC patients, and *BUB1* was significantly associated with living status and clinical stage in TCGA data (Table 1 and Table 2). The analysis results of the remaining genes were shown in Tables S2‐S17. In addition, all nine hub genes were validated to be significantly up‐regulated in ACCs as they were in above three GEO datasets (Figure 7).

**Table 1:** Clinicopathological parameters and *BUB1* expression according to the TCGA database

Parameters	Group	*BUB1* mRNA expression			
		Low(n = 38)	High(n = 39)	X^2^	*P* value
Age (Mean ± SD)		46.63 ± 15.756	46.59 ± 16.106		
Gender	Female	24	24	0.021	1.000
	Male	14	15		
Clinical stage	Ⅰ/Ⅱ	31	15	14.877	.000
	Ⅲ/Ⅳ	7	24		
Recurrence status	No	29	11	2.363	.188
	Yes	7	7		
	Null	2	21		
Living status	Living	34	16	19.841	.000
	Dead	4	23		

**Table 2:** Univariate and multivariate Cox regression analysis of *BUB1* clinical pathologic features according to the TCGA database

Parameters OS	Univariate analysis				Multivariate analysis			
	HR	95% CI		*P*	HR	95% CI		*P*
Age ≥60 vs < 60	1.549	0.677	3.548	.300	0.494	0.207	1.180	.112
Gender Female vs Male	0.986	0.451	2.154	.971				
Clinical stage Ⅰ/Ⅱ vs Ⅲ/Ⅳ	6.467	2.702	15.481	.000	0.208	0.077	0.558	.002
*BUB1* expression Low vs High	9.024	3.094	26.320	.000	5.907	1.920	18.176	.002

## 4 DISCUSSION

In this study, by integrated three expression profiling datasets from GEO, we identified 200 commonly changed DEGs (74 up‐regulated and 126 down‐regulated) in ACCs. These DEGs were further analysed by GO analysis (molecular function, biological process and cellular component). In terms of biological process, the up‐regulated DEGs were mainly enriched in organelle fission and nuclear division, which were typical malignant indicators of histopathological examination. Down‐regulated DEGs were significantly enriched in angiogenesis. For example, *Plk1* is closely related to a series of mitotic events such as centrosome replication, spindle formation, chromosome segregation and cytokinesis and is also related to chromosome stability.^21^ In addition, down‐regulated DEGs were significantly enriched in proteinaceous extracellular matrix. In terms of molecular function, up‐regulated DEGs were significantly enriched in tubulin binding and microtubule binding. It had been reported that *STMN1* regulated microtubule dynamics and participates in the malignant phenotype of cancer cells.^22^ Down‐regulated DEGs were significantly enriched in growth factor activity and growth factor binding. Some studies have shown that the stimulation of growth factors such as insulin‐like growth factors may promote tumour proliferation.^23^ These results can help us to further understand the role of DEGs in the development and progress of ACC. The additional KEGG pathway analysis showed that up‐regulated DEGs were significantly enriched in cell cycle, cell senescence, progesterone‐mediated oocyte maturation, oocyte, p53 signalling pathway and folic acid resistance, further confirmed the important roles of p53 signalling pathway in ACC.^24^

By DEGs PPI network analysis, the hub genes with highest degree of communication were identified, and they are *CCNB1, CDK1, TOP2A, CCNA2, CDKN3, MAD2L1, RACGAP1, BUB1* and *CCNB2. CCNB1*, a key molecule to initiate mitosis, was associated with tumorigenesis and development.^25^ *CCNB2* can promote cell proliferation, migration and invasion in lung adenocarcinoma 26 and hepatocellular carcinoma.^27^ In addition, *CCNB1* and *CCNB2* promoted gastric cancer cell proliferation and tumour growth.^28^ *CDK1* and *TOP2A* played an important role in the regulation of cell cycle and regulated the proliferation of tumours.^29^, ^30^ Glover et al found that *CDK1* was up‐regulated in ACC tissues compared with normal tissues.^31^ *TOP2A* was a potential biomarker for the progression and prognosis of various tumours.^32^ *CCNA2* was found to promote the proliferation of breast cancer.^33^ Xing *et al* found that the expression of *CDKN3* was generally increased in hepatocellular carcinoma tissues and was positively correlated with the pathological stage and differentiation of the tumours.^34^ *MAD2L1* and *BUB1* were important components of mitotic checkpoint complex proteins. High expression of these two genes was related to the poor disease‐free survival of invasive tumours.^35^ *RACGAP1* was found to be highly expressed in colorectal cancer^36^ and breast cancer.^37^

Finally, we evaluated the prognostic value of 9 hub genes by using GEPIA database and found ACC patients with high expression of 9 hub genes (*CCNB1, CDK1, TOP2A, CCNA2, CDKN3, MAD2L1, RACGAP1, BUB1* and *CCNB2*) were significantly associated with worse OS.

In conclusion, using multiple cohort profiling datasets and integrated bioinformatics analysis, we identified hub genes and potential pathways that may be involved in the progress of ACC.

## ETHICS APPROVAL AND CONSENT TO PARTICIPATE

This study has been proved by the Henan University institutional committee.

## CONFLICT OF INTEREST

The authors declare that there is no conflict of interests. No animal or human studies were carried out by the authors for this article.

## AUTHOR CONTRIBUTIONS

XG and LX involved in study concept and design; LX, JG, YG and XG involved in acquisition of data; JG, YG, XM, LZ, HL, ZY and YH involved in analysis and interpretation of data. LX, JG and XG drafted the manuscript; LX, JG and XG involved in critical revision of the manuscript for intellectual content.

## Supporting information

Supplementary Material

## Data Availability

All data generated or analysed during this study are included in this article.
