# Supplementary material for: Identification of hub genes and pathways in adrenocortical carcinoma by integrated bioinformatic analysis
Source: J Cell Mol Med. 2020 Mar 8;24(8):4428–38. doi: 10.1111/jcmm.15102 (PMC7176852; doi:10.1111/jcmm.15102)
Supplement: Supplementary file 1 — Supplementary Material [file JCMM-24-4428-s001.docx]

**Supplementary Materials**

**Figure S1.** Volcano plot of gene expression profiles of DEGs between ACC samples and normal tissues. (A) Volcano plot of GSE10927. (B) Volcano plot of GSE12368. (C) Volcano plot of GSE90713. Red, blue and black color represents relatively high, low and equal expression of genes in the corresponding group, respectively.

**
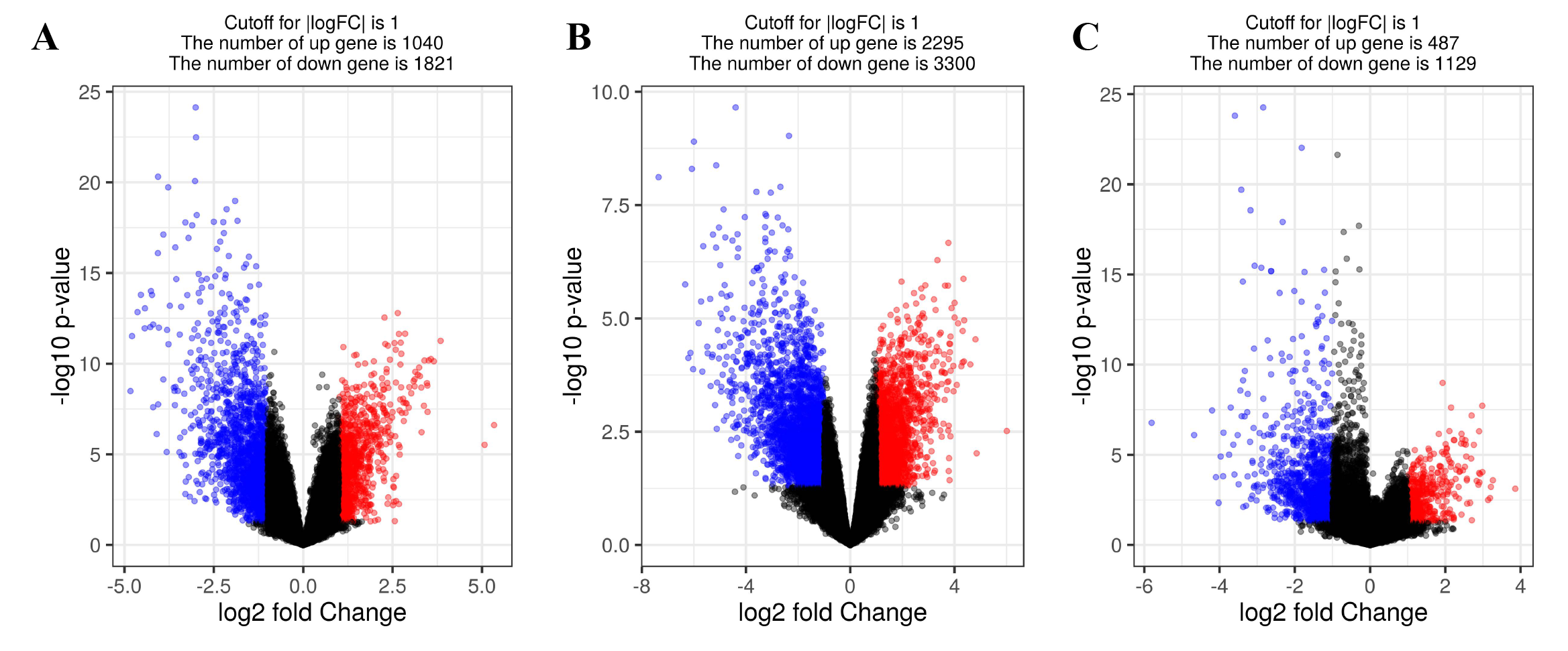
**

**Table S1 Gene ontology analysis of 9 hub genes.**

| Category | GO Number | GO Term | Count | % | P value |
| --- | --- | --- | --- | --- | --- |
| BP | GO:0007094 | mitotic spindle assembly checkpoint | 2 | 0.12 | 0.01 |
| MF | GO:0005524 | ATP binding | 3 | 0.18 | 0.05 |
| KEGG pathway | cfa04914 | Progesterone-mediated oocyte maturation | 6 | 0.36 | 2.99E-10 |
|  | cfa04110 | Cell cycle | 6 | 0.36 | 1.85E-09 |
|  | cfa04114 | Oocyte meiosis | 4 | 0.2442 | 3.89E-05 |
|  | cfa04115 | p53 signaling pathway | 3 | 0.18 | 8.96E-04 |
|  | cfa04914 | Progesterone-mediated oocyte maturation | 6 | 0.36 | 2.99E-10 |

**Table S2. Clinicopathological parameters and *CCNA2* expression according to the TCGA database**

| Parameters | Group | *CCNA2* mRNA expression | | | |
| --- | --- | --- | --- | --- | --- |
|  |  | Low(n=38) | High(n=39) | X^2^ | *P* value |
| Age(Mean±SD) |  | 44.84±15.999 | 48.33±15.675 |  |  |
| Gender | Female | 24 | 24 | 0.021 | 1.000 |
|  | Male | 14 | 15 |  |  |
| Clinical stage | Ⅰ/Ⅱ | 31 | 15 | 14.877 | 0.000 |
|  | Ⅲ/Ⅳ | 7 | 24 |  |  |
| Recurence status | No | 27 | 13 | 0.048 | 1.000 |
|  | Yes | 9 | 5 |  |  |
|  | Null | 2 | 21 |  |  |
| Living status | Living | 34 | 16 | 19.841 | 0.000 |
|  | Dead | 4 | 23 |  |  |

**Table S3. Univariate and multivariate Cox regression analysis of *CCNA2* clinical pathologic features according to the TCGA database**

| Parameters  OS | Univariate analysis | | | |  | Multivariate analysis | | | |
| --- | --- | --- | --- | --- | --- | --- | --- | --- | --- |
|  | HR | 95%CI | | *p* |  | HR | 95%CI | | *p* |
| Age  ≥60 vs <60 | 1.549 | 0.677 | 3.548 | 0.300 |  | 0.533 | 0.222 | 1.277 | 0.158 |
| Gender  Female vs Male | 0.986 | 0.451 | 2.154 | 0.971 |  |  |  |  |  |
| Clinical stage  Ⅰ/Ⅱ vs Ⅲ/Ⅳ | 6.467 | 2.702 | 15.481 | 0.000 |  | 0.197 | 0.073 | 0.535 | 0.001 |
| *CCNA2* expression  Low vs High | 10.217 | 3.498 | 29.849 | 0.000 |  | 7.032 | 2.297 | 21.525 | 0.001 |

**Table S4. Clinicopathological parameters and *CCNB1* expression according to the TCGA database**

| Parameters | Group | *CCNB1* mRNA expression | | | |
| --- | --- | --- | --- | --- | --- |
|  |  | Low(n=38) | High(n=39) | X^2^ | *P* value |
| Age(Mean±SD) |  | 47.74±16.530 | 45.51±15.250 |  |  |
| Gender | Female | 25 | 23 | 0.381 | 0.640 |
|  | Male | 13 | 16 |  |  |
| Clinical stage | Ⅰ/Ⅱ | 29 | 17 | 9.849 | 0.003 |
|  | Ⅲ/Ⅳ | 9 | 24 |  |  |
| Recurence status | No | 25 | 15 | 0.671 | 0.531 |
|  | Yes | 7 | 7 |  |  |
|  | Null | 6 | 17 |  |  |
| Living status | Living | 31 | 19 | 9.128 | 0.004 |
|  | Dead | 7 | 20 |  |  |

**Table S5. Univariate and multivariate Cox regression analysis of *CCNB1* clinical pathologic features according to the TCGA database**

| Parameters  OS | Univariate analysis | | | |  | Multivariate analysis | | | |
| --- | --- | --- | --- | --- | --- | --- | --- | --- | --- |
|  | HR | 95%CI | | *p* |  | HR | 95%CI | | *p* |
| Age  ≥60 vs <60 | 1.549 | 0.677 | 3.548 | 0.300 |  | 0.409 | 0.173 | 0.970 | 0.042 |
| Gender  Female vs Male | 0.986 | 0.451 | 2.154 | 0.971 |  |  |  |  |  |
| Clinical stage  Ⅰ/Ⅱ vs Ⅲ/Ⅳ | 6.467 | 2.702 | 15.481 | 0.000 |  | 0.156 | 0.060 | 0.409 | 0.000 |
| *CCNB1* expression  Low vs High | 4.375 | 1.837 | 10.420 | 0.001 |  | 3.329 | 1.349 | 8.216 | 0.009 |

**Table S6. Clinicopathological parameters and *CCNB2* expression according to the TCGA database**

| Parameters | Group | *CCNB2* mRNA expression | | | |
| --- | --- | --- | --- | --- | --- |
|  |  | Low(n=38) | High(n=39) | X^2^ | *P* value |
| Age(Mean±SD) |  | 47.45±15.656 | 45.79±16.157 |  |  |
| Gender | Female | 23 | 25 | 0.105 | 0.816 |
|  | Male | 15 | 14 |  |  |
| Clinical stage | Ⅰ/Ⅱ | 31 | 15 | 14.877 | 0.000 |
|  | Ⅲ/Ⅳ | 7 | 24 |  |  |
| Recurence status | No | 29 | 11 | 6.018 | 0.024 |
|  | Yes | 5 | 9 |  |  |
|  | Null | 4 | 19 |  |  |
| Living status | Living | 33 | 17 | 15.813 | 0.000 |
|  | Dead | 5 | 22 |  |  |

**Table S7. Univariate and multivariate Cox regression analysis of *CCNB2* clinical pathologic features according to the TCGA database**

| Parameters  OS | Univariate analysis | | | |  | Multivariate analysis | | | |
| --- | --- | --- | --- | --- | --- | --- | --- | --- | --- |
|  | HR | 95%CI | | *p* |  | HR | 95%CI | | *p* |
| Age  ≥60 vs <60 | 1.549 | 0.677 | 3.548 | 0.300 |  | 0.462 | 0.195 | 1.099 | 0.081 |
| Gender  Female vs Male | 0.986 | 0.451 | 2.154 | 0.971 |  |  |  |  |  |
| Clinical stage  Ⅰ/Ⅱ vs Ⅲ/Ⅳ | 6.467 | 2.702 | 15.481 | 0.000 |  | 0.203 | 0.076 | 0.545 | 0.002 |
| *CCNB2* expression  Low vs High | 6.846 | 2.566 | 18.268 | 0.000 |  | 4.347 | 1.526 | 12.380 | 0.006 |

**Table S8. Clinicopathological parameters and *CDK1* expression according to the TCGA database**

| Parameters | Group | *CDK1* mRNA expression | | | |
| --- | --- | --- | --- | --- | --- |
|  |  | Low(n=38) | High(n=39) | X^2^ | *P* value |
| Age(Mean±SD) |  | 44.58±16.710 | 48.59±14.869 |  |  |
| Gender | Female | 26 | 22 | 1.183 | 0.349 |
|  | Male | 12 | 17 |  |  |
| Clinical stage | Ⅰ/Ⅱ | 32 | 14 | 18.679 | 0.000 |
|  | Ⅲ/Ⅳ | 6 | 25 |  |  |
| Recurence status | No | 28 | 12 | 0.771 | 0.512 |
|  | Yes | 8 | 6 |  |  |
|  | Null | 2 | 21 |  |  |
| Living status | Living | 34 | 16 | 19.841 | 0.000 |
|  | Dead | 4 | 23 |  |  |

**Table S9. Univariate and multivariate Cox regression analysis of *CDK1* clinical pathologic features according to the TCGA database**

| Parameters  OS | Univariate analysis | | | |  | Multivariate analysis | | | |
| --- | --- | --- | --- | --- | --- | --- | --- | --- | --- |
|  | HR | 95%CI | | *p* |  | HR | 95%CI | | *p* |
| Age  ≥60 vs <60 | 1.549 | 0.677 | 3.548 | 0.300 |  | 0.528 | 0.219 | 1.274 | 0.155 |
| Gender  Female vs Male | 0.986 | 0.451 | 2.154 | 0.971 |  |  |  |  |  |
| Clinical stage  Ⅰ/Ⅱ vs Ⅲ/Ⅳ | 6.467 | 2.702 | 15.481 | 0.000 |  | 0.240 | 0.087 | 0.664 | 0.006 |
| *CDK1* expression  Low vs High | 10.712 | 3.660 | 31.354 | 0.000 |  | 6.486 | 2.071 | 20.315 | 0.001 |

**Table S10. Clinicopathological parameters and *CDKN3* expression according to the TCGA database**

| Parameters | Group | *CDKN3* mRNA expression | | | |
| --- | --- | --- | --- | --- | --- |
|  |  | Low(n=38) | High(n=39) | X^2^ | *P* value |
| Age(Mean±SD) |  | 45.66±16.313 | 47.54±15.498 |  |  |
| Gender | Female | 25 | 23 | 0.381 | 0.640 |
|  | Male | 13 | 16 |  |  |
| Clinical stage | Ⅰ/Ⅱ | 31 | 15 | 14.877 | 0.000 |
|  | Ⅲ/Ⅳ | 7 | 24 |  |  |
| Recurence status | No | 27 | 13 | 0.903 | 0.506 |
|  | Yes | 8 | 6 |  |  |
|  | Null | 3 | 20 |  |  |
| Living status | Living | 33 | 17 | 15.813 | 0.000 |
|  | Dead | 5 | 22 |  |  |

**Table S11. Univariate and multivariate Cox regression analysis of *CDKN3* clinical pathologic features according to the TCGA database**

| Parameters  OS | Univariate analysis | | | |  | Multivariate analysis | | | |
| --- | --- | --- | --- | --- | --- | --- | --- | --- | --- |
|  | HR | 95%CI | | *p* |  | HR | 95%CI | | *p* |
| Age  ≥60 vs <60 | 1.549 | 0.677 | 3.548 | 0.300 |  | 0.490 | 0.205 | 1.168 | 0.107 |
| Gender  Female vs Male | 0.986 | 0.451 | 2.154 | 0.971 |  |  |  |  |  |
| Clinical stage  Ⅰ/Ⅱ vs Ⅲ/Ⅳ | 6.467 | 2.702 | 15.481 | 0.000 |  | 0.194 | 0.072 | 0.523 | 0.001 |
| *CDKN3* expression  Low vs High | 7.484 | 2.799 | 20.009 | 0.000 |  | 4.928 | 1.737 | 13.982 | 0.003 |

**Table S12. Clinicopathological parameters and *MAD2L1* expression according to the TCGA database**

| Parameters | Group | *MAD2L1* mRNA expression | | | |
| --- | --- | --- | --- | --- | --- |
|  |  | Low(n=38) | High(n=39) | X^2^ | *P* value |
| Age(Mean±SD) |  | 21 | 27 | 1.599 | 0.244 |
| Gender | Female | 17 | 12 |  |  |
|  | Male | 27 | 19 | 3.992 | 0.063 |
| Clinical stage | Ⅰ/Ⅱ | 11 | 20 |  |  |
|  | Ⅲ/Ⅳ | 24 | 16 | 0.035 | 1.000 |
| Recurence status | No | 8 | 6 |  |  |
|  | Yes | 6 | 17 |  |  |
|  | Null | 31 | 19 | 9.128 | 0.004 |
| Living status | Living | 7 | 20 |  |  |
|  | Dead | 21 | 27 | 1.599 | 0.244 |

**Table S13. Univariate and multivariate Cox regression analysis of *MAD2L1* clinical pathologic features according to the TCGA database**

| Parameters  OS | Univariate analysis | | | |  | Multivariate analysis | | | |
| --- | --- | --- | --- | --- | --- | --- | --- | --- | --- |
|  | HR | 95%CI | | *p* |  | HR | 95%CI | | *p* |
| Age  ≥60 vs <60 | 1.549 | 0.677 | 3.548 | 0.300 |  | 0.514 | 0.213 | 0.364 | 0.137 |
| Gender  Female vs Male | 0.986 | 0.451 | 2.154 | 0.971 |  |  |  |  |  |
| Clinical stage  Ⅰ/Ⅱ vs Ⅲ/Ⅳ | 6.467 | 2.702 | 15.481 | 0.000 |  | 0.142 | 0.055 | 0.364 | 0.000 |
| *MAD2L1* expression  Low vs High | 3.767 | 1.588 | 8.937 | 0.003 |  | 3.053 | 1.239 | 7.523 | 0.015 |

**Table S14. Clinicopathological parameters and *RACGAP1* expression according to the TCGA database**

| Parameters | Group | *RACGAP1* mRNA expression | | | |
| --- | --- | --- | --- | --- | --- |
|  |  | Low(n=38) | High(n=39) | X^2^ | *P* value |
| Age(Mean±SD) |  | 24 | 24 | 0.021 | 1.000 |
| Gender | Female | 14 | 15 |  |  |
|  | Male | 30 | 16 | 11.508 | 0.001 |
| Clinical stage | Ⅰ/Ⅱ | 8 | 23 |  |  |
|  | Ⅲ/Ⅳ | 28 | 12 | 3.276 | 0.108 |
| Recurence status | No | 6 | 8 |  |  |
|  | Yes | 4 | 19 |  |  |
|  | Null | 33 | 17 | 15.813 | 0.000 |
| Living status | Living | 5 | 22 |  |  |
|  | Dead | 24 | 24 | 0.021 | 1.000 |

**Table S15. Univariate and multivariate Cox regression analysis of *RACGAP1* clinical pathologic features according to the TCGA database**

| Parameters  OS | Univariate analysis | | | |  | Multivariate analysis | | | |
| --- | --- | --- | --- | --- | --- | --- | --- | --- | --- |
|  | HR | 95%CI | | *p* |  | HR | 95%CI | | *p* |
| Age  ≥60 vs <60 | 1.549 | 0.677 | 3.548 | 0.300 |  | 0.492 | 0.206 | 1.179 | 0.112 |
| Gender  Female vs Male | 0.986 | 0.451 | 2.154 | 0.971 |  |  |  |  |  |
| Clinical stage  Ⅰ/Ⅱ vs Ⅲ/Ⅳ | 6.467 | 2.702 | 15.481 | 0.000 |  | 0.183 | 0.069 | 0.486 | 0.001 |
| *RACGAP1* expression  Low vs High | 6.685 | 2.517 | 17.755 | 0.000 |  | 4.417 | 1.589 | 12.277 | 0.004 |

**Table S16. Clinicopathological parameters and *TOP2A* expression according to the TCGA database**

| Parameters | Group | *TOP2A* mRNA expression | | | |
| --- | --- | --- | --- | --- | --- |
|  |  | Low(n=38) | High(n=39) | X^2^ | *P* value |
| Age(Mean±SD) |  | 25 | 23 | 0.381 | 0.640 |
| Gender | Female | 13 | 16 |  |  |
|  | Male | 29 | 17 | 8.571 | 0.005 |
| Clinical stage | Ⅰ/Ⅱ | 9 | 22 |  |  |
|  | Ⅲ/Ⅳ | 26 | 14 | 0.275 | 0.749 |
| Recurence status | No | 8 | 6 |  |  |
|  | Yes | 4 | 19 |  |  |
|  | Null | 32 | 18 | 12.242 | 0.001 |
| Living status | Living | 6 | 21 |  |  |
|  | Dead | 25 | 23 | 0.381 | 0.640 |

**Table S17. Univariate and multivariate Cox regression analysis of *TOP2A* clinical pathologic features according to the TCGA database**

| Parameters  OS | Univariate analysis | | | |  | Multivariate analysis | | | |
| --- | --- | --- | --- | --- | --- | --- | --- | --- | --- |
|  | HR | 95%CI | | *p* |  | HR | 95%CI | | *p* |
| Age  ≥60 vs <60 | 1.549 | 0.677 | 3.548 | 0.300 |  | 0.441 | 0.183 | 1.060 | 0.067 |
| Gender  Female vs Male | 0.986 | 0.451 | 2.154 | 0.971 |  |  |  |  |  |
| Clinical stage  Ⅰ/Ⅱ vs Ⅲ/Ⅳ | 6.467 | 2.702 | 15.481 | 0.000 |  | 0.175 | 0.068 | 0.452 | 0.000 |
| *TOP2A* expression  Low vs High | 5.760 | 2.298 | 14.440 | 0.000 |  | 4.390 | 1.644 | 11.720 | 0.003 |
